# The Effect of Real-Time Medication Monitoring-Based Digital Adherence Tools on Adherence to Antiretroviral Therapy and Viral Suppression in People Living With HIV: A Systematic Literature Review and Meta-Analysis

**DOI:** 10.1097/QAI.0000000000003449

**Published:** 2024-07-09

**Authors:** Takondwa Charles Msosa, Iraseni Swai, Rob Aarnoutse, Tobias F. Rinke de Wit, Kennedy Ngowi, Chisomo Msefula, Marriott Nliwasa, Marion Sumari-de Boer

**Affiliations:** a Department of Global Health, Amsterdam UMC, location University of Amsterdam, Amsterdam, the Netherlands;; b Amsterdam Institute for Global Health and Development, Amsterdam, the Netherlands;; c Helse Nord Tuberculosis Initiative, Kamuzu University of Health Sciences, Blantyre, Malawi;; d Kilimanjaro Clinical Research Institute, Moshi, Tanzania;; e Radboud University Medical Center, Department of Pharmacy, Research Institute for Medical Innovation, Nijmegen, the Netherlands; and; f Institute of Public Health, Kilimanjaro Christian Medical University College, Moshi, Tanzania.

**Keywords:** HIV, viral load, ART, adherence, real-time medication monitoring, digital, adherence tools

## Abstract

**Background:**

Universal antiretroviral therapy (ART) has led to improved treatment outcomes in persons living with HIV. Adherence to ART is required to achieve viral suppression. Real-time medication monitoring (RTMM)–based digital adherence tools (DATs) could be effective in improving ART adherence and viral suppression in persons living with HIV.

**Objectives:**

The primary and secondary objectives of this review were to assess the effect of RTMM-based DATs on improving ART adherence and viral load suppression.

**Methods:**

We searched MEDLINE, Embase, and Global Health for publications published through October 11, 2022. Narrative synthesis and random effects meta-analyses were conducted to synthesize the results.

**Results:**

Of 638 papers identified, 8 were included. Six studies were randomized controlled trials (RCTs), and 2 were cohort studies. Two studies, an RCT in China (mean adherence: 96.2% vs 89.1%) and a crossover cohort study in Uganda (mean adherence: 84% vs 93%), demonstrated improved ART adherence. No studies demonstrated improved viral suppression. In the meta-analyses, we estimated that RTMM-based digital adherence tools had a statistically insignificant small positive effect on ART adherence and viral suppression with a standardized mean difference of 0.1922 [95% CI: −0.0268 to 0.4112, *P*-value: 0.0854] and viral suppression with an odds ratio of 1.3148 [95% CI: 0.9199 to 1.8791, *P*-value: 0.1331].

**Conclusions:**

Our meta-analyses found that RTMM-based DATs did not have a significant effect on ART adherence and viral suppression. However, due to few published studies available, heterogeneity of target populations, intervention designs, and adherence measurement instruments, more data are required to provide conclusive evidence.

## Abstract 2

Supplemental Digital Content is Available in the Text.

## INTRODUCTION

HIV/AIDS continues to be a disease of major public health importance worldwide. By the end of 2021, approximately 38.4 million people were living with HIV (PLHIV) of whom 88% were on antiretroviral therapy (ART).^1^ Initiation of ART upon HIV diagnosis has greatly contributed to reduced HIV-associated morbidity and mortality^2,3^; however, the effectiveness of ART depends on how well patients adhere to their prescribed medication.^4,5^ Therefore, adherence to antiretroviral treatment is critically important, both at the individual and population levels. Suboptimal adherence to an ART regimen is associated with a greater risk of developing resistance to antiretroviral agents and increases the risk of HIV-related morbidity and mortality, as well as progression to AIDS.^6,7^ Furthermore, poor ART adherence leads to increased costs for healthcare systems due to increased incidence of hospitalization, management of opportunistic infections, treatment failure, drug resistance, and use of more expensive second-line and third-line ART.^8^ In the early stages of ART, it was recommended that an adherence of 95% was required to achieve viral suppression.^6,9^

However, with the advent of more potent regimens in recent years, a medication adherence of 80% or more is sufficient to achieve viral suppression.^6,10–12^ Various factors have been attributed to nonadherence to ART, which include stigma, depression, fear of HIV status disclosure, forgetfulness, medication boredom, and a busy schedule.^13–18^ Therefore, due to the high penetration of mobile devices and networks, various digital interventions have been developed to assist PLHIV address adherence challenges.^19–22^ Among these interventions is the use of real-time medication monitoring (RTMM)–based digital adherence tools (DATs). RTMMs make use of pill boxes that send cellular signals to a web-based server in real time when the pill box is opened.^23^ The opening of the box is also recorded in real time on a server and is called a medication intake event. The patient's or carer's mobile phone number and medication intake time are registered in an online database or server to enable the sending of short message service (SMS) reminders before their medication intake time or triggered when the box is not opened in the recorded or prescribed medication intake time. Additionally, information kept on the server can be used by healthcare providers to monitor patients' adherence patterns and provide RTTM-informed interventions such as customized adherence feedback or “just-in-time counseling.”^23^

Multiple studies have shown that RTMM-based DATs are acceptable among PLHIV and are feasible in resource-limited settings.^24–28^ However, the major concern with RTMM-based DATs is the possibility of unwanted HIV status disclosure due to the triggered reminder SMSs and concerns of invasion of privacy.^29^ Although most studies have reported good feasibility of the intervention, some have reported technical challenges such as poor network as barriers to effective implementation of RTMM-based interventions.^24,26^

Therefore, in this review, we sought to synthesize evidence on the effect of RTTM-based DATs in improving ART adherence and viral load suppression in PLHIV. The primary objective was to determine the effect of RTMM-based DATs in improving ART adherence in PLHIV. The secondary outcome was to determine the effect of RTMM-based DATs in 3 improving viral load suppression in PLHIV.

## METHODS

### Search Strategy and Selection Criteria

Using the Ovid platform, we searched MEDLINE, Embase, and Global Health for studies published through October 11, 2022 (see Supplemental Digital Content 1, http://links.lww.com/QAI/C294). We also searched the following clinical trial registries: ClinicalTrials.gov, the International Clinical Trials Registry Platform (ICTRP), and the Pan African Clinical Trials Registry (PACTR) for completed trials. The database search was conducted on October 11, 2022, and there were no restrictions on the publication dates. The inclusion criteria included randomized controlled trials (RCTs) and cohort studies in PLHIV on ART, studies that used RTMM as part of a digital adherence intervention, and studies that reported medication adherence and/or viral load as their outcomes. RTMM-based interventions were defined as interventions that used RTMM in combination with at least 1 of the following interventions: customized or RTMM-informed adherence feedback, RTMM-informed home visits, triggered SMS reminders, just-in-time counseling, or an RTMM-informed phone call. Therefore, the intervention had to include an “RTMM-triggered action.” We excluded cross-sectional and case-control studies, as well as studies that used RTMM solely as a measurement instrument and not as part of an intervention to improve either ART adherence or viral load suppression. We have reported the results of this review according to the recommendations by the Preferred Reporting Items for Systematic Reviews and Meta-Analyses (see Supplemental Digital Content 2, http://links.lww.com/QAI/C295).^30^

### Study Selection and Data Extraction

The online platform Covidence was used for the selection and screening of studies that were identified in the database searches.^31^ Two reviewers, TCM and IS, independently conducted the title, abstract, and full-text screening. Disputes between the 2 authors were resolved through consensus. Relevant study characteristics and variables were extracted independently by TCM and IS using a custom data extraction tool that collected the following information: author, year of publication, country, title, study design, population, intervention, comparator, outcomes, follow-up period, and results. Inconsistencies and disputes in the data extraction were again resolved through consensus between the 2 reviewers. Study authors of the included studies were contacted to provide additional information where necessary. In addition, for multiple-arm trials, only data from eligible arms were included.

Furthermore, for studies that reported adherence using the median and interquartile range (IQR), we converted to means and standard deviations in scenarios in which we did not have access to the raw data upon request.

For our meta-analyses, we extracted the following additional information for the primary outcome: number of participants in the intervention and control arms, unadjusted mean adherence in the intervention and control arms, and the standard deviations of the unadjusted mean adherence in the intervention and control arms. For the secondary outcome, we extracted the following additional information: number of participants in the intervention and control arms, respectively, and the number of participants with suppressed viral loads in the intervention and control arms respectively.

### Quality Assessment

To assess the risk of bias and study quality, the Cochrane Risk of Bias Tool Version 2 (RoB 2)^32^ was used for RCTs and the Newcastle Ottawa Scale^33^ was used for cohort studies. For the RCTs, the risk of bias for each item was evaluated at 3 levels: high, low, and some concerns. If studies were evaluated to have a high overall risk of bias, the studies were considered to be of low quality.

### Data Analysis

A narrative synthesis was conducted, and the results were summarized with emphasis on the study populations, intervention design, comparators, and outcomes. The outcomes of ART adherence and/or viral load were described as reported in the included studies. We only incorporated RCTs in our meta-analyses to ensure high methodological quality.

Random effects models were employed to calculate summary outcome measures and 95% confidence intervals (CIs) for the primary and secondary outcomes. We employed the standardized mean difference (SMD) approach to pool estimates for the cumulative mean adherence outcome and pooled odds ratios (ORs) for the proportion with suppressed viral loads. Viral load suppression was defined as <50 copies/mL.

In scenarios where studies had multiple adherence measurement methods, the following predetermined order for the most accurate method was utilized: electronic monitoring, pill counting, pharmacy refill counts, and self-report. This was adapted from a systematic review by Wang et al.^34^

We assessed heterogeneity between studies using the I^2^ statistic. Heterogeneity in both the primary and secondary outcomes was explored by stratifying by intervention target population and follow-up period as these were deemed to be probable moderators. We hypothesized that the intervention effect might vary between ART-naive and ART-experienced participants and that it might also vary depending on the length of time the participants used the intervention, in which we categorized the intervention duration into more than 6 months and less than or equal to 6 months.

Sensitivity analyses on the primary outcome involved restricting our analysis to studies that measured adherence by RTMM only and only including full RCTs to check whether our estimates were consistent.

Finally, publication bias was explored using funnel plots and the Egger regression test. Symmetry in the funnel plot was an indication for the absence of publication bias and a *P*-value < 0.05 in the Egger test was considered to be strong evidence for the presence of publication bias. All analyses were conducted in R (version 4.2.1.) using the meta package (version 6.5).^35^

### Ethical Considerations

This review used published data and therefore ethical approval was not obtained.

## RESULTS

### Study Selection

**FIGURE 1.**
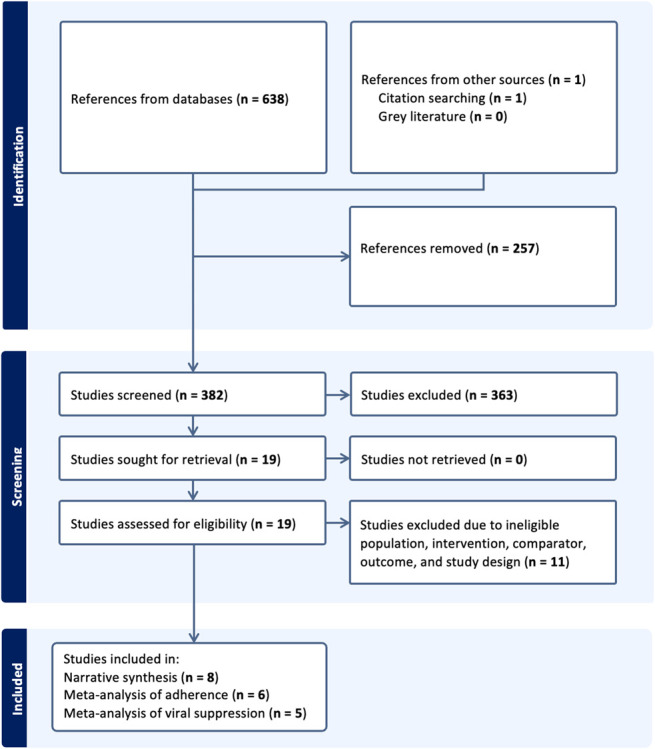
PRISMA flowchart.

The database search yielded a total of 638 studies, and 1 study was discovered through manual citation tracking. Two hundred fifty-seven studies were eliminated as duplicates, and a total of 382 studies were subjected to title and abstract screening, of which 19 were deemed relevant and subjected to full-text screening. Eleven studies were excluded because they did not meet the inclusion criteria: 7 due to ineligible interventions, 3 due to ineligible study designs, and 1 due to ineligible outcomes (see Supplemental Digital Content 3, http://links.lww.com/QAI/C296). Therefore, a total of 8 studies were included in the qualitative synthesis, with 6 contributing to the meta-analysis on adherence and 5 contributing to the meta-analysis on HIV viral suppression. Figure 1 is a summary of the included papers.

### Study Characteristics

The 8 eligible studies were published between 2014 and 2022: 6 studies were conducted in Africa,^19–21,36–38^ 1 in China,^22^ and 1 in the United States.^29^ Four studies were RCTs: 2 were pilot RCTs and 2 were cohort studies. All eligible studies included adult PLHIV who were on antiretroviral therapy, and no studies included children or adolescents. Furthermore, 1 study in Uganda included ART-naive pregnant women who were initiating ART^38^; 1 study in South Africa included ART-naive participants with clinically advanced disease or a CD4 count of <350 cells/μL^19^; 1 cohort study included PLHIV on second-line ART^20^; and another study from China included a mixture of ART-naive and ART-experienced PLHIV who were deemed to be at high risk of virological failure.^22^ Similarly, the intervention design was diverse in the included studies, combining RTMM with RTMM-triggered SMS reminders and/or RTMM plus customized/RTMM-informed adherence feedback. The follow-up time of the included studies varied from 3 months to a maximum of 12 months.

More information on study characteristics can be seen in Table 1.

**TABLE 1.** Characteristics of Included Studies (SOC)

Author	Study Design	Number of Enrolled Participants	Population	Intervention/Exposure	Comparator	Outcomes	Follow-Up Time	Result
Pellowski et al,^29^ USA	Pilot RCT	41	Adult PLHIV on ART with adherence of 90% or less adherence and on an ART regimen of 2 dose times a day	RTMM + just-in-time adherence counseling	Standard pillbox and alarm	Adherence by unannounced pill count	3 mo	No difference in mean adherence between study arms (F [1, 37] = 1.07, *P* = 0.31, d = 0.33)
Orrell et al,^19^ South Africa	RCT	230	ART-naive participants with clinically advanced disease or a CD4 count of <350 cells/μL	RTMM + triggered SMS + SOC	SOC + RTMM	1. Adherence by pharmacy refill count 2. Adherence by self-report 3. Adherence by RTMM 4. Plasma viral load	12 mo	Median adherence by RTMM was 82.1% (IQR, 56.6%–94.6%) in the intervention arm and 80.4% (IQR, 52.8%–93.8%) in the control arm. there was no difference in the odds of virological failure (>40 copies/mL) in the intervention arm (OR, 0.77; CI: 0.41 to 1.4; *P* = 0.393) compared with the control arm
Sabin et al,^22^ China	RCT	120	Adult PLHIV owning a mobile phone and at risk for poor adherence	RTMM + standard adherence counseling + RTMM feedback counseling + triggered reminder SMS	RTMM + standard adherence counseling	1. Adherence by RTMM 2. Plasma viral load <50 copies/mL	6 months for adherence outcome and 9 months for viral load	Mean adherence was significantly higher in intervention vs control arm: 96.3% (SD = 5.8%) vs 88.9% (SD = 14.65) (*P* < 0.001). Proportions with undetectable viral load were similar between intervention vs. control subjects, 58/62 (93.6%) vs 54/55 (98.2%), respectively (*P* = 0.218)
Haberer et al,^36^ Uganda	Pilot RCT	63	Adult PLHIV and their social supporters owning a personal cell phone with reliable reception	1. Scheduled SMS + real-time adherence monitoring (“scheduled SMS arm”) 2. Triggered SMS + real-time adherence monitoring (“triggered SMS arm”)	Real-time adherence monitoring only (called the “control”): Study participants in this arm received no SMS reminders	1. Adherence measured by RTMM 2. HIV RNA <100 c/mL	9 mo	The intervention was not effective in improving ART adherence compared with the standard of care; mean difference of −0.7% (*P* = 0.90). No statistically significant differences in HIV RNA suppression (<100 c/mL) were seen among study arms (*P* = 0.14)
Sumari-de Boer et al,^21^ Tanzania	RCT	249	Suboptimal-adherent adult PLHIV on antiretroviral treatment for at least 6 mo	1. Scheduled reminder SMS + prompt SMS + structured adherence feedback 2. RTMM + reminder SMS + structured adherence feedback	SOC	1. Adherence by pharmacy refill count 2. Plasma viral load	12 mo	Mean pharmacy refill adherence was 87.9% (SD = 12.9) for the control arm, 89.6% (SD = 12.9) for the SMS arm, and 90.6% (SD = 10.8) for the intervention arm, *P* = 0.36. No difference in proportion virologically suppressed at 6 months at <1000 copies/mL and <20 copies/mL, respectively
Sabin et al,^38^ Uganda	RCT	133	Adult ART-naive pregnant women initiating ART in Uganda	RTMM + triggered SMS + data-informed counseling	SOC + RTMM	1. Proportion of participants achieving ≥95% adherence during the final 30 days of the intervention period 2. Proportion ≥95% adherence over 3 mo 3. Mean adherence over 3 mo 4. Mean adherence during the final 30 d	3 mo	No difference in mean adherence throughout 3 months, 63.4 vs 62.1 (*P* = 0.82)
Evans et al,^20^ South Africa	Cohort	764	Adult PLHIV failing ART with a viral load of ≥400 copies/mL on second-line ART	Intensive adherence counseling + real-time medication monitor + triggered SMS reminder	1. Standard adherence counseling 2. Intensive adherence counseling	Alive, in care, and suppressed: viral suppression was defined as a repeat viral load <400 copies/mL	6 mo	The intervention was not effective in improving viral load suppression: adjusted relative risk (ARR) of 1.06 (95% CI, 0.75–1.49) when compared with standard adherence counseling, and ARR of 1.17 (0.83–1.66) when compared with intensive adherence counseling
Haberer et al^37^, Uganda	Crossover cohort study	112	Adult ART-naive participants commencing treatment	Real-time electronic adherence monitoring device + follow-up: sustained (≥48 h) interruptions)	Standard medication event monitoring system	1. Adherence measured by MEMS for the first 6 mo 2. Adherence measured by RTMM for the second 6 mo	6 mo	Mean adherence among 112 participants increased from 84% to 93% and remained elevated for 6 mo (*P* < 0.001) No difference in viral load suppression (6% versus 7%, *P* = 0.48)

### Risk of Bias in Studies

We assessed the risk of bias for RCTs using the Cochrane RoB 2 for each study outcome. All eligible studies had some concerns in the overall assessment (see Supplemental Digital Content 4, http://links.lww.com/QAI/C297). Furthermore, we assessed the study quality for the cohort studies using the Newcastle Ottawa Scale. The highest score was of a crossover cohort study from Uganda with a score of 7 out of 10. A cohort study from South Africa scored 5 out of 9.

### Narrative Synthesis

There was diversity in the target populations, intervention designs, follow-up periods, and outcome measures. In terms of the effect of the intervention on ART adherence, only 2 studies demonstrated a positive intervention effect. An RCT in China which investigated the effect of RTMM plus RTTM-triggered SMS reminders and RTMM-informed customized adherence feedback in both ART-naive and ART-experienced patients showed a positive intervention effect with a cumulative mean adherence of 96.2% in the intervention arm compared with 89.1% in the standard-of-care (SOC) arm.^22^ In addition, a crossover cohort study in Uganda demonstrated an improvement in ART adherence from 84% to 93% after participants switched to an RTMM-based adherence intervention after using a more standard medication event monitoring system called MEMS.^37^ However, the results of this crossover cohort should be interpreted with caution due to the change in the measurement instrument, from standard medication event monitoring systems to real-time medication monitors.

In terms of viral load suppression, none of the studies demonstrated either a positive or negative intervention effect. One cohort study in South Africa, which sought to determine the effect of the intervention in comparison with standard adherence counseling in PLHIV on second-line ART, showed that there was no statistically significant difference in viral load suppression (<400 copies/mL) with an adjusted RR of 1.06 (95% CI: 0.75 to 1.49).^20^ Similarly, all the included trials demonstrated no intervention effect in improving viral load suppression when compared with the SOC across diverse participant populations, intervention designs, and intervention periods.^19,21,22,36,38^ Another important observation is that 2 trials disclosed providing generous financial and material incentives in all study arms to reduce loss to follow-up rates.^19,22^

A summary of the results from individual studies can be seen in Table 1.

### Meta-Analyses

**FIGURE 2.**
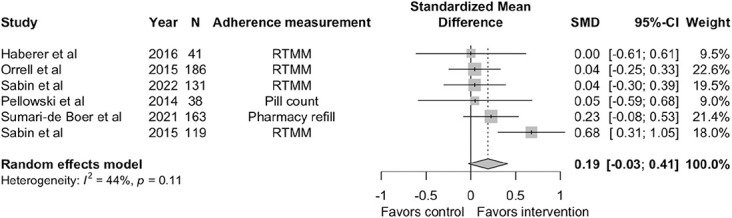
Forest plot of standardized mean difference in adherence.

Six trials reported on adherence.^19,21,22,29,36,38^ The results of the meta-analysis of the primary outcome showed that the intervention had a statistically insignificant small positive effect on ART adherence with a standardized mean difference (SMD) of 0.1922 [95% CI: −0.0268 to 0.4112, *P*-value: 0.0854] when comparing the intervention and control arms. Furthermore, the directionality of the effect in all the individual studies was positive, favoring the intervention, except 1 study which showed a null effect. However, there was substantial heterogeneity in the included studies with an I^2^ of 44% (*P* = 0.11). Figure 2 is the forest plot of the meta-analysis.

**FIGURE 3.**
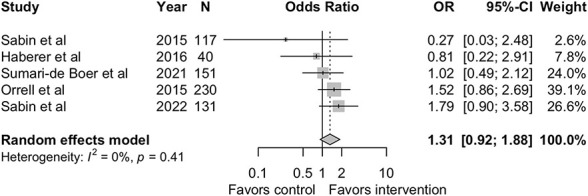
Effect of RTMM-based DATs on viral load suppression.

Five studies reported on the secondary outcome.^19,21,22,36,38^ There was no statistically significant difference in proportion with suppressed viral loads in the intervention arm compared with the SOC with a pooled OR of 1.3148 (95% CI: 0.9199 to 1.8791, *P* = 0.1331). In addition, 3 studies favored the intervention^19,21,38^ and 2 studies did not favor the intervention.^22,36^ There was no evidence of heterogeneity in the included studies: I^2^ = 0% (*P* = 0.4055). Figure 3 is a forest plot of this meta-analysis.

### Subgroup Analyses

In the subgroup analysis, heterogeneity across studies in relation to the primary and secondary outcome could not be explained by follow-up time and target population as can be seen in Table 2.

**TABLE 2.** Effectiveness of RTMM-Based DATs on ART Adherence and Viral Load Suppression

Subgroup	Number of Trials	SMD or OR (95% CI)	Q Value	*P* for Heterogeneity
Subgroup analysis of ART adherence				
Follow-up time			0.44	0.5072
>6 mo	3	0.1143 (−0.0847 to 0.3133)*		
≤6 mo	3	0.2781 (−0.1633 to 0.7195)*		
Target population			2.28	0.1309
ART experienced	3	0.3553 (−0.0014 to 0.7120)*		
ART naive	3	0.0373 (−0.1702 to 0.2448)*		
Subgroup analysis of viral load suppression				
Target population			0.09	0.7662
>6 mo	3	1.2401 (0.8113 to 1.8955)†		
≤6 mo	2	0.9420 (0.1618 to 5.4845)†		
Time on ART			1.30	0.2549
ART experienced	2	0.8044 (0.2956 to 2.1891)†		
ART naive	3	1.5104 (0.9956 to 2.2913)†		

* Standardized mean difference in adherence.

† OR of viral load suppression.

### Sensitivity Analysis

For the adherence outcome, a sensitivity analysis was conducted to determine the consistency of the results when only full RCTs and not pilot RCTs were included in the meta-analysis. The results remained consistent with the primary analysis: SMD of 0.2343 [−0.0431 to 0.5117]. Furthermore, when only studies that measured adherence using RTMMs were meta-analyzed, the results were also consistent with the primary analysis: SMD of 0.2006 [−0.1269 to 0.5282].

### Publication Bias

**FIGURE 4.**
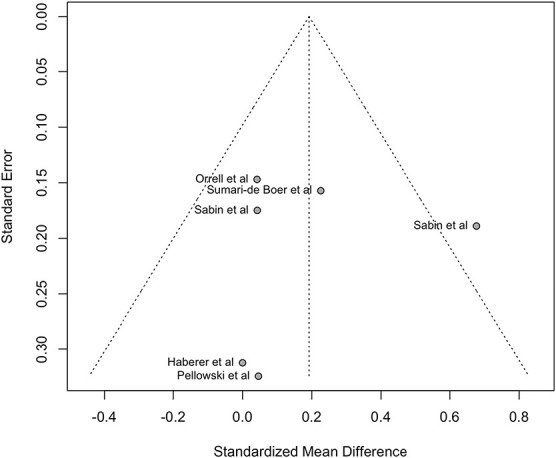
Funnel plot on the effect of RTMM-based DATs on ART adherence.

The funnel plot for the primary outcome (adherence) among the randomized trials can be seen in Figure 4. The number of studies included in the plot is low, which makes it difficult to judge whether the asymmetry in the plot is due to publication bias, chance, methodological quality, or heterogeneity in the studies. However, the Egger regression test of funnel plot asymmetry showed no evidence of publication bias (*P*-value = 0.9144).

## DISCUSSION

Our systematic literature review and meta-analyses showed that RTMM-based DATs did not significantly improve ART adherence or viral suppression in PLHIV, although this conclusion is limited by the low number of studies published examining their effect or impact. These findings were consistent across patient populations, intervention designs, and intervention implementation periods.

To the best of our knowledge, this is the first systematic review and meta-analysis that specifically sought to synthesize evidence on the effect of RTMM-based DATs on adherence to ART and viral suppression in PLHIV. Several systematic reviews have been conducted on the effect of digital health interventions on ART adherence and viral suppression in PLHIV.

For example, a systematic review by Wang et al^34^ suggested that digital health interventions had a small positive effect on adherence (Cohen d = 0.25; 95% CI: 0.05 to 0.46; *P* = 0.01). However, a subgroup analysis in the review by Wang et al,^34^ which included 2 trials on RTMM-based DATs, showed no positive intervention effect in improving ART adherence or viral suppression, consistent with the results of our meta-analysis.

There are several important limitations in the evidence included in this review. First, there were a limited number of published studies included in both the narrative synthesis and meta-analyses. Furthermore, all studies that contributed to the meta-analyses had some concern over the overall risk of bias assessment, which could negatively affect the validity of our results. Another important limitation that we encountered is the heterogeneity in the design of the intervention and SOC in the publications. In terms of the design, there were diverse combinations of RTMMs with customized adherence feedback, home visits, caregiver participation, and triggered SMS reminders. Furthermore, the minimum and maximum follow-up durations ranged from 3 to 12 months. All these factors put together could influence the effect of the intervention in the various studies. In addition, the use of RTMMs to measure medication adherence in the control arms of some RCTs may have concealed the actual effect of RTMM-based interventions compared with real SOC due to the Hawthorne effect. This is because participants in the comparator/SOC arms could have positively changed their adherence behavior due to the possession of the device and their awareness of being monitored even though the devices were not used as an intervention. The provision of generous financial and material incentives to all study participants in the intervention and control arms in some studies to mitigate attrition^19,22^ could have attenuated the effect size as participants in the control arm may have been motivated to be more adherent to their ART because of the incentives.

Furthermore, these incentives could influence the external validity or generalizability of the intervention as this may not truly reflect real-world use in HIV care and treatment clinics in which the provision of incentives is not standard practice. Another important limitation is the lack of participant and clinic staff blinding in the RCTs due to the nature of the RTMM-based intervention, which could have negatively affected the internal validity of the trials due to observer influence or bias. Finally, since the majority of the databases that were searched indexed publications written in English, we did not include studies that were published in other languages leading to selection bias.

However, our review has several strengths. First, our search strategy was tailored to RTMM-based DATs, which allowed for clear and focused search strategies and analyses to provide meaningful conclusions on this intervention, which would not have been possible in an overarching review of digital health interventions due to their broader scope. Second, in the meta-analyses of the primary and secondary outcomes, we only included RCTs, which greatly enhanced the quality and credibility of our results. Third, we standardized effect measures of both the primary and secondary outcomes minimizing the effect of heterogeneous cut-offs used in the analysis and reporting of individual studies.

Finally, our results were consistent in the sensitivity analyses of the primary outcome providing evidence of the robustness of our results and assumptions.

To provide conclusive evidence on the effect of this intervention, we suggest that investigators should take into consideration the following points to improve the internal and external validity of future trials. First, future trials should consider PLHIV with high viral loads due to poor adherence to ART as the target population as this is ultimately the population that might benefit the most from this intervention. Furthermore, future trials should not only confine study populations to adults but should consider involving children and adolescents as they are a demographic group that is prone to poor adherence and viral nonsuppression. Third, when designing trials, investigators should ensure that control arms reflect the true SOC so that the true effect of RTMM-based interventions can be assessed. Fourth, we suggest that investigators should consider using combinations of methods to measure adherence to improve the accuracy of the measurement of this outcome.^39^ For example, pharmacological measures of adherence, electronic adherence monitors, self-reported adherence, or pharmacy refill counts can be combined with viral load measurements to improve the accuracy of measurements and enable investigators to differentiate poor adherence from ART resistance.^39^ Last, the DAT intervention has been reported to have several technical limitations such as poor network coverage, limited battery life, and inconsistent sending of triggered SMS reminders^19,21,22^; therefore, in future trials, investigators should ensure that these challenges are mitigated to improve fidelity.

## CONCLUSIONS

In conclusion, our systematic review and meta-analyses summarized evidence on whether RTMM-based DATs affect ART adherence or viral load suppression. In our meta-analyses, we found that RTMM-based DATs did not significantly improve ART adherence or viral load suppression in PLHIV. However, due to the heterogeneity of population groups, intervention designs, and adherence measurement instruments, the results of this review and meta-analysis need to be interpreted with caution and further inquiry into this intervention needs to be undertaken to identify appropriate target populations, intervention designs, measurement instruments of adherence, follow-up periods, and outcome measures. Therefore, more high-quality studies need to be conducted to determine the efficacy of RTMM-based DATs in PLHIV.

## Supplementary Material
