# Supplementary material for: The Effect of Real-Time Medication Monitoring-Based Digital Adherence Tools on Adherence to Antiretroviral Therapy and Viral Suppression in People Living With HIV: A Systematic Literature Review and Meta-Analysis
Source: J Acquir Immune Defic Syndr. 2024 Jul 9;96(5):411–20. doi: 10.1097/QAI.0000000000003449 (PMC11236270; doi:10.1097/QAI.0000000000003449)
Supplement: Supplementary file 1 [file qai-96-411-s001.pdf]

Key words

1. Real time medication monitor
2. Antiretroviral therapy
3. Adherence
4. Viral load

Medline search strategy (248 hits)

1	exp Reminder Systems/ or real-time medication monitor*.mp. or electronic adherence monitor*.mp. or real-time adherence monitor*.mp. or real-time monitoring*.mp. or digital adherence monitor*.mp. or medication event monitor*.mp.
2	exp Antiretroviral Therapy, Highly Active/ or exp Anti-HIV Agents/ or exp Anti-Retroviral Agents/
3	exp Patient Compliance/ or exp Medication Adherence/ or adherence.mp. or compliance.mp. or self-efficacy.mp.
4	exp Viral Load/ or viral load.mp. or virological suppression.mp. or viral suppression.mp
5	3 or 4
6	1 and 2 and 5

Embase search strategy (317 hits)

1	Reminder Systems.mp. or exp reminder system/ or real-time medication monitor*.mp. or electronic adherence monitor.mp or real-time adherence monitor*.mp. or real-time monitoring*.mp. or digital adherence monitor*.mp. or exp medication event monitoring system/ or medication event monitor*.mp.
2	antiretroviral therapy.mp. or exp antiretroviral therapy/ or Anti-Retroviral Agents.mp. or exp antiretrovirus agent/ or Anti-HIV Agents.mp. or exp anti human immunodeficiency virus agent/ or highly active antiretroviral therapy.mp. or exp highly active antiretroviral therapy/
3	medication adherence.mp. or exp medication compliance/ or compliance.mp. or adherence.mp. or self-efficacy.mp. or medication compliance.mp. or medication self-efficacy.mp.
4	viral load*.mp. or virus load.mp. or virological suppression*.mp.
5	3 or 4
6	1 and 2 and 5

Global Health search strategy (73 hits)

1	(Reminder Systems* or real-time medication monitor* or electronic adherence monitor or real-time adherence monitor* or real-time monitoring or digital adherence monitor* or medication event monitoring system or medication event monitor*).mp. [mp=abstract, title, original title, heading words, cabicodes words]

2	(antiretroviral therapy or Anti-Retroviral Agent*).mp. or antiretrovirus agent*/ or Anti-HIV Agent*.mp. or anti human immunodeficiency virus agent.mp. or highly active antiretroviral therapy.mp. [mp=abstract, title, original title, heading words, cabicodes words]
3	(compliance or adherence or self-efficacy or medication compliance or medication adherence or medication self-efficacy).mp. [mp=abstract, title, original title, heading words, cabicodes words]
4	(viral load* or virus load or virological suppression).mp. [mp=abstract, title, original title, heading words, cabicodes words]
5	3 or 4
6	1 and 2 and 5
