# Supplementary material for: The Effect of Real-Time Medication Monitoring-Based Digital Adherence Tools on Adherence to Antiretroviral Therapy and Viral Suppression in People Living With HIV: A Systematic Literature Review and Meta-Analysis
Source: J Acquir Immune Defic Syndr. 2024 Jul 9;96(5):411–20. doi: 10.1097/QAI.0000000000003449 (PMC11236270; doi:10.1097/QAI.0000000000003449)
Supplement: Supplementary file 3 [file qai-96-411-s003.docx]

A total of 11 studies were excluded. Reasons for exclusion can be seen in the table below:

| **First Author** | **Year of publication** | **Title** | **Reason for exclusion** |
| --- | --- | --- | --- |
| Bien-Gund | 2021 | Financial incentives and real-time adherence monitoring to promote daily adherence to HIV treatment and viral suppression among people living with HIV: a pilot study | **Ineligible intervention**: Intervention included receiving of daily ($2.55) lottery-based financial reward for opening the electronic bottle and an incentive of $100 for achieving viral suppression at 3 months.  Control; half of the participants in the control arm used the electronic bottle but didn’t get incentives. |
| Ukwe C.V | 2010 | Self-reported adherence to HAART in south-eastern Nigeria is related to patients' use of pill box | **Ineligible study design**: This was a prospective, observational study, that assessed predictors of adherence to a combination antiretroviral. There was no intervention group. |
| Escobar-Viera C | 2020 | The Florida mobile health adherence project for people living with HIV (FL-mAPP): Longitudinal assessment of feasibility, acceptability, and clinical outcomes | **Ineligible intervention**: Intervention included the use of SMS reminders only or the use of the mHealth Adherence Application (full app). Participants submitted a dashboard-generated adherence report during follow-up visits |
| Wu, Albert W | 2006 | A randomized trial of the impact of a programmable medication reminder device on quality of life in patients with AIDS. | **Ineligible outcome:** The study evaluated the impact on quality of life (QOL) of a medication reminder device. The intervention group received the Disease Management Assistance System (DMAS), a prompting device that verbally reminds patients of medication times and electronically records doses, and a monthly 30-minute adherence educational session. Controls received education only. |
| Haberer J.E. | 2010 | Real-time adherence monitoring for HIV antiretroviral therapy | **Ineligible study design:** The study compared different adherence methods. All Participants had adherence monitoring monthly with a visual analog scale (VAS), self-reported recall of doses missed over the previous 3 days, and unannounced home-based pill count, as well as electronic monitoring with the medication event monitoring system (MEMS). |
| Andrade, | 2005 | A programmable prompting device improves adherence to highly active antiretroviral therapy in HIV-infected subjects with memory impairment. | **Ineligible intervention:** The intervention was the Disease Management Assistance System (DMAS) device, combined with monthly adherence counseling. Control subjects received only adherence counseling. |
| Fairley C.K | 2003 | Randomized trial of an adherence programme for clients with HIV | **Ineligible intervention:** This was a randomized step wedge (pre, post) design in which the intervention included an education programme, individualized planning of regimens, and use of the reminder devices. |
| Chung | 2011 | A randomized controlled trial comparing the effects of counseling and alarm device on HAART adherence and virologic outcomes | **Ineligible intervention:** Interventions included counseling (three counseling sessions around HAART initiation), alarm (pocket alarm carried for 6 months, counseling plus alarm, and neither counseling nor alarm. |
| DeFulio A | 2021 | Smartphone-based incentives for promoting adherence to antiretroviral therapy: A randomized controlled trial | **Ineligible intervention:** This was **a** smartphone-based adherence intervention in which participants submitted video selfies of medication consumption and received incentives for a valid video. |
| Pang Y. | 2020 | Preliminary Effects of a Mobile Interactive Supervised Therapy Intervention on People Living With HIV: Pilot Randomized Controlled Trial | Ineligible intervention: The intervention didn’t address adherence in real-time. It included the use of a Medication Event Monitoring System (MEMS)—and a mobile app—MIST (Mobile Interactive Supervised Therapy). Adherence reports were downloaded and discussed with participants during follow-up visits. |
| Saberi P | 2015 | Correlation between use of antiretroviral adherence devices by HIV-infected youth and plasma HIV RNA and self-reported adherence | **Ineligible study design:** The study assessed associations of device use with viral suppression and self-reported adherence. Participants used either of the following devices; use of labels, calendars, pillboxes, beepers, timers, medication event monitoring caps (MEMS), programmable wristwatches, and diaries. |
