# Supplementary material for: The Effect of Real-Time Medication Monitoring-Based Digital Adherence Tools on Adherence to Antiretroviral Therapy and Viral Suppression in People Living With HIV: A Systematic Literature Review and Meta-Analysis
Source: J Acquir Immune Defic Syndr. 2024 Jul 9;96(5):411–20. doi: 10.1097/QAI.0000000000003449 (PMC11236270; doi:10.1097/QAI.0000000000003449)
Supplement: Supplementary file 4 [file qai-96-411-s004.docx]

# Risk of bias assessments

Figure 1 is a summary of the risk of bias assessment for the adherence outcome in the randomised controlled trials.


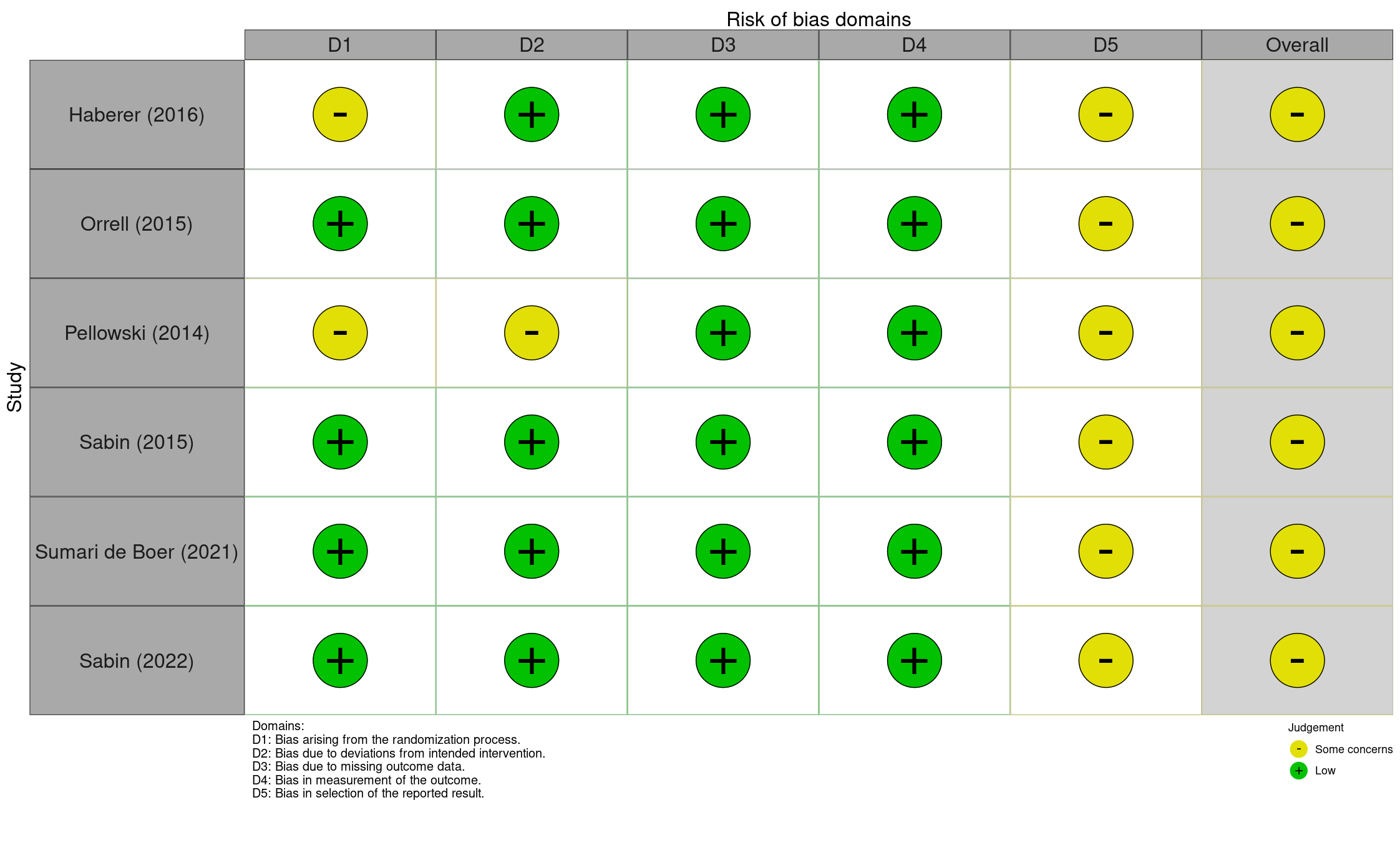


Figure 1: Risk of bias assessment for adherence outcome

Figure 2 is a summary of the risk of bias assessment for the viral load suppression outcome in the randomised controlled trials.


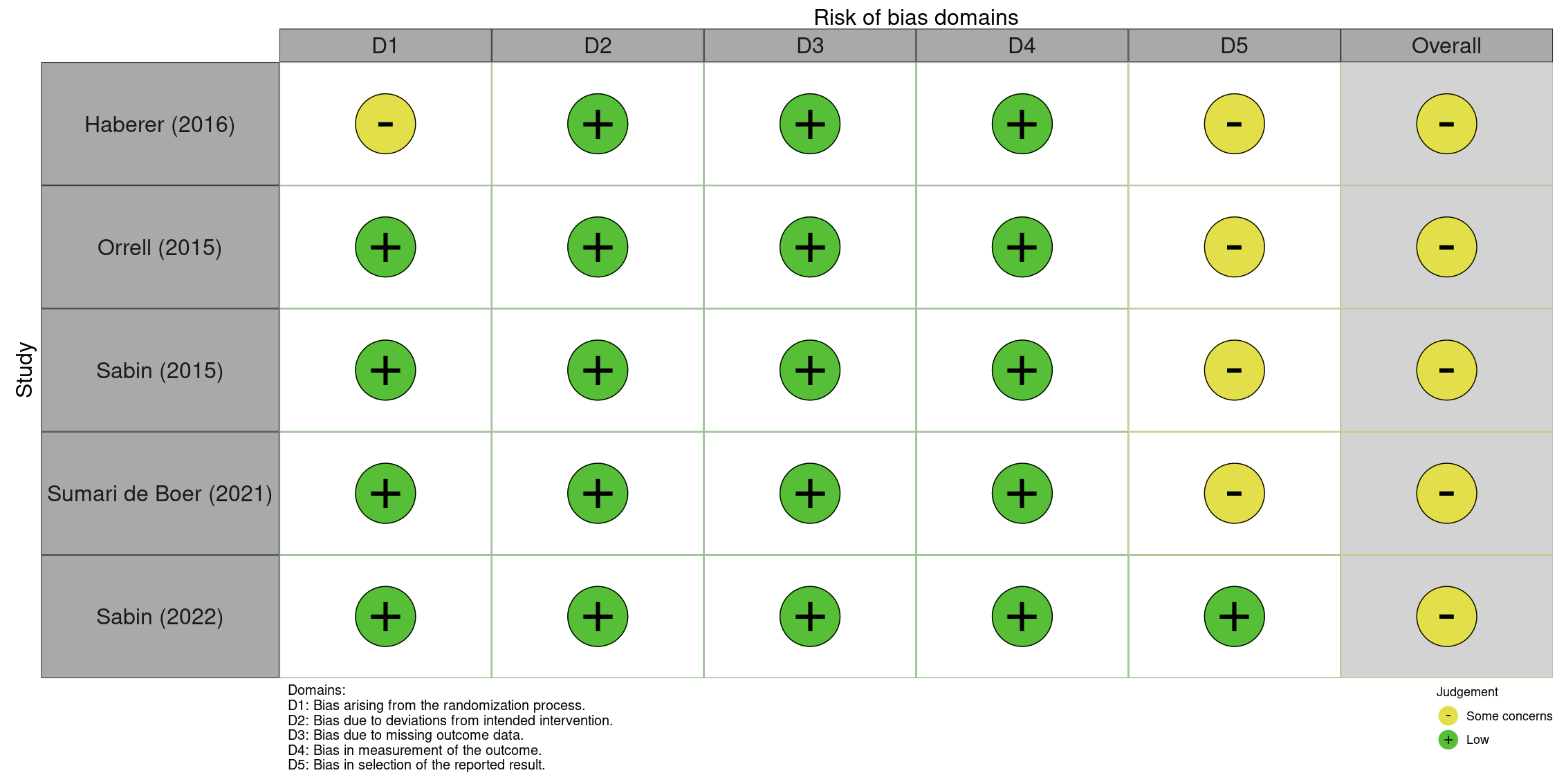


Table 2 is the quality assessments of the cohort studies using the Newcastle-Ottawa Scale

Table 2: Quality assessment of cohort studies

| Study (year) | Outcome | A. Selection (maximum of four stars) | | | | B. Comparability (maximum of two stars) | C. Outcome (maximum of three stars) | | | Total (maximum of nine stars) |
| --- | --- | --- | --- | --- | --- | --- | --- | --- | --- | --- |
|  |  | 1. Representativeness of the exposed cohort | 2. Selection of the non-exposed cohort | 3. Ascertainment of exposure | 4. Demonstration that outcome of interest was not present at start of study | 1. Comparability of cohort on the basis of the design or analysis | 1. Assessment of outcome | 2. Was follow-up long enough for outcomes to occur | 3. Adequacy of follow-up of cohorts |  |
| Jessica E. Haberer (2017) | Adherence | ★ | ★ | ★ | ☆ | ★★ | ☆ | ★ | ★ | 7 |
| Jessica E. Haberer (2017) | Viral load | ★ | ★ | ★ | ☆ | ★★ | ☆ | ★ | ★ | 7 |
| Denise Evans (2016) | Viral load | ☆ | ★ | ☆ | ☆ | ★★ | ★ | ★ | ☆ | 5 |
